# Imerslund-Gräsbeck syndrome in a 25-month-old Italian girl caused by a homozygous mutation in *AMN*

**DOI:** 10.1186/1824-7288-39-58

**Published:** 2013-09-17

**Authors:** Gianpaolo De Filippo, Domenico Rendina, Vincenzo Rocco, Teresa Esposito, Fernando Gianfrancesco, Pasquale Strazzullo

**Affiliations:** 1 Servizio di Endocrinologia Pediatrica, AORN “Gaetano Rummo”, Benevento, Italy; 2 Dipartimento di Medicina Clinica e Chirurgia, Università degli Studi Federico II, Naples, Italy; 3 Laboratorio di Patologia Clinica, AORN “Gaetano Rummo”, Benevento, Italy; 4 Istituto di Genetica e Biofisica “Adriano Buzzati-Traverso” CNR, Naples, Italy; 5 Actual address: Service d’Endocrinologie et Diabétologie Pédiatrique, Hôpitaux Universitaires Paris Sud, Hôpital Bicêtre, Le Kremlin Bicêtre, France

**Keywords:** Imerslund-Gräsbeck syndrome, Anemia, Ethnicity, Mutation screening, Amnionless

## Abstract

Imerslund-Gräsbeck syndrome is a rare autosomal recessive disorder, characterized by vitamin B12 deficiency due to selective malabsorption of the vitamin and usually results in megaloblastic anemia appearing in childhood. It is responsive to parenteral vitamin B12 therapy.

The estimated prevalence (calculated based on Scandinavian data) is less than 6:1,000,000. However, many cases may be misdiagnosed.

When there is reasonable evidence to suspect that a patient suffers from IGS, a new and straightforward approach to diagnosis is mutational analysis of the appropriate genes. We report for the first time the case of a girl of Italian ancestry with IGS genetically confirmed by the detection of a homozygous missense mutation in the *AMN* gene (c.208-2 A > G).

## Background

Imerslund-Gräsbeck syndrome or juvenile megaloblastic anemia (IGS, OMIM #261100) is a rare autosomal recessive disorder independently described by Imerslund [1] and Gräsbaeck [2] in 1960. It is characterized by vitamin B12 (cobalamin, cbl) deficiency due to selective malabsorption of the vitamin and usually results in megaloblastic anemia appearing in childhood (but not immediately after birth). It is responsive to parenteral vitamin B12 therapy. Mild proteinuria is frequently but not always present.

Mutations in the cubilin (*CUBN*, OMIM # 602997) [3] and amnionless (*AMN*, OMIM # 605799) genes [4] account for most cases of IGS, whereas mutations in the gastric intrinsic factor gene (*GIF*, OMIM # 609342), which cause intrinsic factor deficiency (IFD), phenocopy genuine IGS [5].

Approximately 300 IGS cases have been published worldwide, with new cases predominantly appearing in eastern Mediterranean countries. The estimated prevalence (calculated based on Scandinavian data) is less than 6:1,000,000 [6]. However, many cases may be misdiagnosed.

IGS patients usually present within the first 5 years of life with megaloblastic anemia, proteinuria and decreased serum vitamin B12 levels, which are sometimes accompanied by neurological symptoms [7]. Failure to-thrive is also associated with IGS [8,9].

Diagnosing IGS is a time-consuming and often inconclusive procedure based primarily on excluding other causes of Cbl deficiency, which are many and vary with age (e.g., malnutrition, lack of Intrinsic Factor, consumption of Cbl by parasitic worms or bacterial overgrowth of the small intestine, exocrine pancreatic insufficiency, ileitis terminalis, congenital defects of *CUBN, AMN, GIF, TCNI and TCNII*, and pharmacotherapy) [10].

Until recently, absorption tests with radiocobalt-labeled cobalamin were routinely used to study patients with megaloblastic anemia and related conditions. Schilling’s urinary excretion technique was the most popular method [11]. When there is reasonable evidence to suspect that a patient suffers from IGS, a new and straightforward approach to diagnosis is mutational analysis of the appropriate genes.

## Case presentation

We report the case of a 25-month-old Italian girl who presented with failure to-thrive and megaloblastic anemia. She was born at 38 weeks of gestation by cesarean section from parents of southern Italian origin who were not aware of any consanguinity. Her birth weight was 2.950 kg, and length was 49 cm. The perinatal and neonatal periods were uneventful. She was breast-fed until 6 months and received only vitamin D supplementation. Her ponderal growth was normal (at the 50^th^ percentile) until the third month of life, when it slowly but progressively began to decelerate from the 50^th^ percentile to the 5^th^ percentile by 21 months. Her statural growth was consistently at the 25^th^ percentile. Her psychomotor development was normal for her age.

At 21 months, she began to lose her appetite, and an obstinate constipation appeared, partially responsive to an osmotic laxative. Over the course of the next three months, she lost weight (−0.200 kg) and then began vomiting once daily for the next week.

At admission, she appeared tired and pale. Her weight was 9.370 kg, length was 85 cm (25^th^ percentile) and cranial circumference was 49 cm (75^th^ percentile). Her heart rate was 120 bpm, blood pressure was 80/50 mmHg, and her temperature was 36.6°C. Her chest was normal to auscultation. Her abdomen was soft, and her liver was not palpable. Her skinfold was normal with dry skin. There were no dysmorphic features. The neurological findings were strictly normal.

The hematological parameters indicated macrocytic anemia hemoglobin 7.8 g/dl, hematocrit 23.9, RBC 1.99 cells/mcL, MCH 39.2 pg, MCV 120.1 fl, reticulocytes 0.7%, WBC 5,980/mcL, platelets 276.000/mcL and reduced serum Cbl level (<100 pg/ml, n.v. 211-911) in the presence of normal serum folate levels (13.2 ng/L). A peripheric smear showed the presence of neutrophilic granulocytes with an augmented volume and hypersegmentated core. All other biochemical indexes were normal (liver,kidney and thyroid function, inflammatory markers, malabsorbtion and celiac disease markers). The urinary excretion of total protein and albumin was normal.

Treatment with parenteral vitamin B12 was initiated (500 μg/day I.M. for 5 days) and resulted in rapid improvement of symptomatology, with recovery from vomiting and anorexia.

At a three-year follow-up appointment, the girl showed normal somatic development, with weight and height at the 50^th^ percentile and normal psychomotor development on a bimestral administration of vitamin B12 (1,000 μg I.M.) regimen. Her hematological parameters were stably normalized.

**Figure 1:**
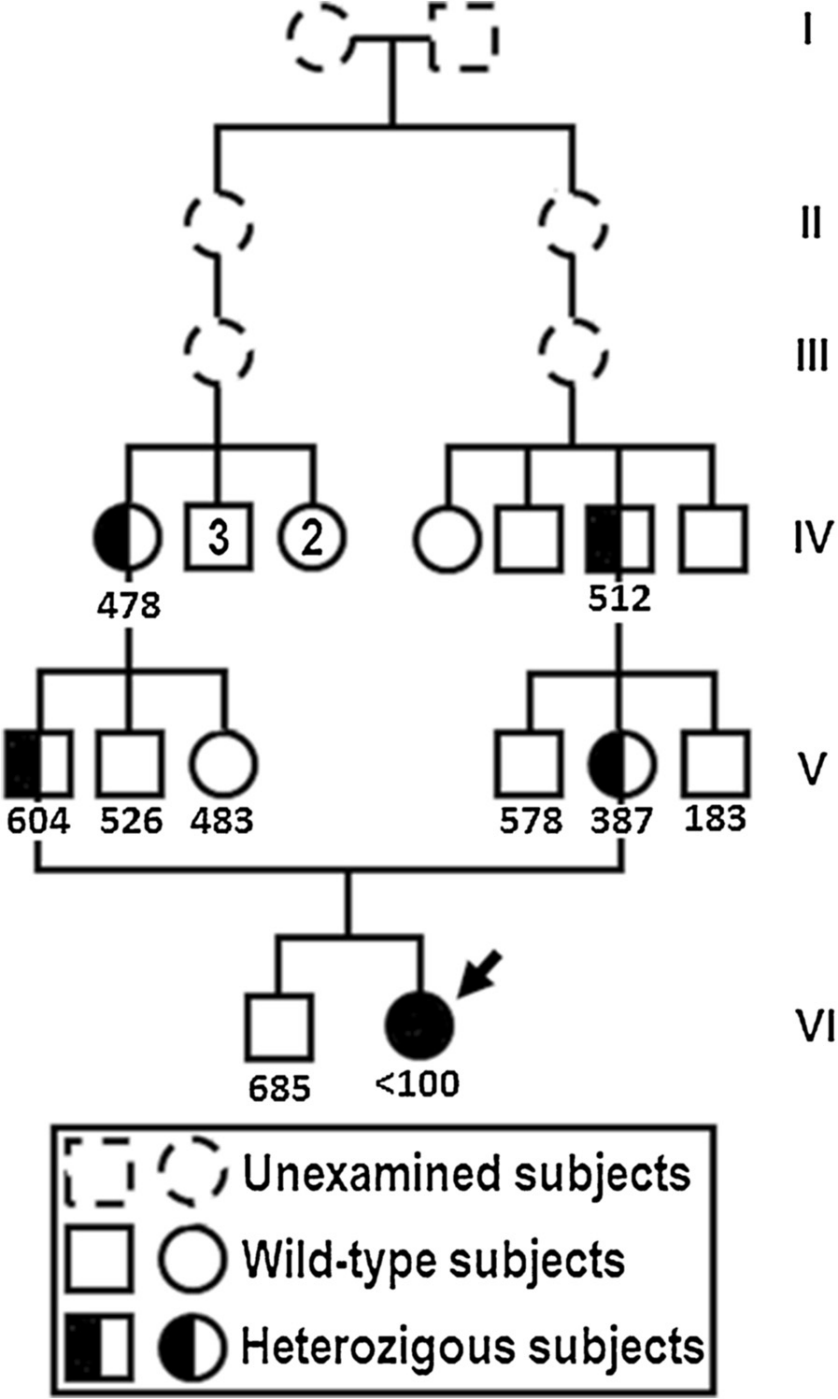
**Pedigree of the patient.** Cobalamin levels are indicated (pg/ml; n.v. 211-911). Heterozygous subjects have normal levels of cobalamin.

To substantiate a clinical diagnosis of IGS, we assessed the familial history of the index patient. A more accurate analysis of the genealogic tree revealed the presence of consanguinity (Figure 1). Thus, after obtaining written informed consent, we proceeded with genetic testing.

All coding regions including intron/exon boundaries of the *AMN* and *CUBN* genes were analyzed by direct sequencing. We identified a heterozygous missense variation, c.1165C > A, in exon 11 of the CUBN gene, which causes the substitution of threonine for proline at position 389 of the CUBN protein. This change has been reported as a polymorphism (rs1801224) in the dbSNP database [11] with a minor allele frequency of 0.45.

Mutational analysis of the *AMN* gene identified the presence of a previously described mutation (c.208-2 A > G) [12] in the splice acceptor site of exon 4; the patient was homozygous for this mutation. Segregation analysis revealed that the parents were both heterozygous for the AMN mutation. This change was absent in 600 unrelated subjects from the same geographical area.

To determine if this mutation results in the skipping of exon 4, causing aberrant splicing of the transcript, as previously described [4], mRNA analysis was performed. mRNA derived from lymphocytes of the father of the index patient was reverse transcribed, amplified with specific primers and sequenced. Two bands were observed, corresponding to transcripts derived from both canonical and alternative splicing. In control mRNA, only the canonical transcript was present.

## Conclusions

We report for the first time the case of a girl of Italian ancestry with IGS genetically confirmed by the detection of a homozygous missense mutation in the *AMN* gene. The condition is rare and if in some cases (i.e. in patients with mild proteinuria) is easier to suspect IGS, in some patients the first symptoms could be vague and it could be more difficult to suspect the disease. Early diagnosis is important and life-long treatment with vitamin B12 is necessary.

In ethnic groups in wich there is a low frequency of consanguineous marriages ad reasonably a lower prevalence of autosomal recessive disorders as IGS, diagnosis should be suspected (and thus mutational analysis performed) only after excluding other common causes of failure to thrive, general malabsorption and megaloblastic anemia. The absence of well-known causes of failure-to-thrive, such as recurrent infections or gastrointestinal complaints, suggests that the metabolic disturbances caused by an isolated cobalamin decificiency as seen in IGS can elicit a failure to thrive [8,9].

*The CUBN* and *AMN* genes encode the two subunits (cubilin and amnionless) of the cobalamin-intrinsic factor of the ileal mucosa [13]. The cubilin-amnionless complex is called cubam and is considered to be essential for intestinal cobalamin uptake, renal protein reabsorption and early rodent embryogenesis [14].

The reported AMN mutation (a.208-2 A > G) has been described in people of Sephardic Jewish or Turkish descent as well as in Arabic families from Jordan. One case from the USA had Hispanic roots, but, based on the patients’ name, the individual was judged to be of Jewish ancestry, and one case from Spain was without detailed ethnic information. The occurrence of this particular mutation in patients originating mainly from the eastern Mediterranean area suggested that c.208-2A > G may be a founder mutation. Given that genetic analysis for newly diagnosed IGS cases is complex, it has been suggested that AMN c.208-2A > G should be considered first when dealing with patients originating from Turkey, Jordan, Spain, or Tunisia or with an ethnic Sephardic background [15]. The current case represents the first time that this mutation has been described in a patient of Caucasian ancestry.

Concerning treatment issues, the vitamin B12 deficiency is first corrected by giving intramuscular injections of cobalamin and is recommended that these injections are then repeated regularly for the rest of the patient’s life. Several therapeutic regimens have been proposed: 1000 μg of hydroxycobalamin i.m. daily for 10 days then 1000 μg i.m. once a month [6] or 1000 μg i.m. weekly for 1 month, then 1000 μg i.m. every three or six months [16]. Successful treatment of IGS with 1 mg of vitamin B12, orally administered at 2-week intervals, has been reported [17]. In view of the accumulating evidence that subclinical deficiency of cobalamin may contribute to the development of atherosclerosis, dementia and osteoporosis [18,19] and considered that cobalamin is non-toxic, it is suggested that patients receive a higer dose of cobalamin than necessary, rather than an insufficient dose [6]. In our case, we decided to taper the dose according to clinical and laboratory response and the outcome was strongly favorable on long term with the administration of 1000 μg of vitamin B12 i.m. every two months.

Our findings indicate that IGS should be considered when diagnosing children with megaloblastic anemia and failure to thrive. Diagnosis should be confirmed genetically, first focusing the study on genes suspected on the basis of ethnicity and successively, if indicated, expanding the research to other potentially involved genes. Genetic confirmation substantiates clinical findings and the necessity of adequate lifelong therapy.

## Informed consent

Written informed consent was obtained from the patient’s parents and all studied subjects for publication of this Case Report and any accompanying images. A copy of the written consent is available for review by the Editor-in-Chief of this journal.

## Competing interests

The authors declare that they have no competing interests.

## Authors’ contributions

GDF, DR and VR made the diagnosis and insure the diagnostic work-up. TE and FG performed the genetic study. PS coordinated the diagnostic work-up. GDF, DR and PS wrote the manuscript. All authors read and approved the final manuscript.
